# Additional qualifications of trainees in specialist training programs in Australia

**DOI:** 10.1186/s12909-019-1686-8

**Published:** 2019-07-05

**Authors:** Daniel Thompson, Colin Thompson, Natasha Nassar, Annette Katelaris

**Affiliations:** 1 St. Vincent’s Hospital Melbourne, 41 Victoria Parade, Fitzroy, Melbourne, Victoria 3065 Australia; 2 Port Macquarie Eye Centre, 35 Ackroyd Street, Port Macquarie, New South Wales 2444 Australia; 3 Children’s Hospital at Westmead Clinical School, University of Sydney, Level 2, Charles Perkins Centre D17, Camperdown, NSW 2006 Australia; 4 Sydney Medical School, University of Sydney, Rm 213B Edward Ford Building A27, Sydney, New South Wales 2006 Australia

**Keywords:** Postgraduate education, Medical education, Specialist training, Surgical training, Registrar

## Abstract

**Background:**

In Australia, the number of medical graduates per year has increased at a greater rate than the increase in the number of specialist training places. Consequently, competition for training positions is intensifying. There is anecdotal evidence to suggest that medical graduates are acquiring additional qualifications to compete with their peers Stevenson 2017 (https://insightplus.mja.com.au/2017/36/specialty-training-places-the-other-looming-crisis/). Our study investigates this phenomenon of additional credentialing and demonstrates the number and type of postgraduate and research qualifications obtained by specialists in training in Australia. This is the first study to assess the number and type of credentials acquired by registrars in each specialty and to provide insight into differences between specialities.

**Methods:**

Information on specialists in training was obtained through the Medicine in Australia: Balancing Employment and Life (MABEL) survey conducted between 2008 and 2014. The number of any additional qualifications and specific PhD, Master’s degree, postgraduate diploma/certificate and research degrees from medical school were assessed for each specialist training scheme in the database.

**Results:**

Overall, 995 registrars representing 13 specialties were included. Just under a third (30.4%) completed a research-based degree during their medical degree and almost half (46.7%) of specialist registrars obtained further qualifications after completing medicine. A significantly higher proportion of ophthalmology (78.6%) and paediatric (67.5%) registrars, and a lower percentage of emergency medicine (36.7%) registrars, held postgraduate qualifications. Overall, 2.4% of registrars held a PhD and 10.1% held a Master’s degree. A higher percentage of either PhD or Master’s was held by ophthalmology (64.3%) and surgical (30.6%) trainees and a lower percentage by anaesthetics (6.3%) and physician trainees (7.9%). Postgraduate diplomas or certificates were most common among paediatric (41.2%) and obstetrics and gynaecology (25.6%) registrars.

**Conclusion:**

This is the first study to investigate the additional qualifications of specialists in training in Australia. Almost half of specialists in training surveyed (46.7%) have completed some form of additional study, whether it is a PhD, Master’s, postgraduate diploma/certificate or research degree from medical school. Trainees of specialist training schemes are more qualified than specialists who trained in the past Aust Fam Physician 32:92-4, 2003.

## Background

Specialist training in Australia is conducted through the 15 specialist medical colleges [1]. The specialist colleges set the number of training positions they will provide each year and the selection criteria for trainees [2]. Each college has their own application process with the positions usually determined on the basis of an interview, referee’s assessment, and curriculum vitae [3]. The minimum eligibility is a completed medical degree. Given the disproportionate number of increasing medical graduates and unmatched number of training positions, there is anecdotal evidence that many potential trainees are undertaking additional activities such as pre-specialisation work and pursuing further qualifications, research and other skills courses in order to increase their competitiveness for selection. There is clear motivation for candidates to obtain further qualifications to better their chances for selection, the colleges make CV scoring guides available for candidates and most give points for additional qualifications. The weighting of points to qualifications varies from speciality to speciality and may be a reason for variation in types of qualifications obtained by speciality trainees of different craft groups [4]. Pre-specialisation work, especially additional qualifications which are not mandatory for clinical practice, increase the cost of, and frequently the time required to complete specialist training, and may come at the cost of gaining clinical or surgical experience.

In Australia, the number of domestic medical graduates has increased by 230% from 2005 to 2015 [5] outstripping the 206% increase in total number of advanced training positions (other than general practice) over the same period [6, 7]. The increase in the number of training places has been inconsistent across specialities and may partly account for the differences in extra credentials obtained between specialty registrars. For example, the number of training places in surgery over this timeframe has only increased by 130% [6, 7].

The rationale of this study was to investigate the nature and extent of qualifications obtained by current Australian speciality trainees. It is the first study to examine this characteristic of trainees, and the phenomenon of over-credentialing with qualifications not specifically required for clinical practice.

## Methods

Data were ascertained for both new and continuing specialist registrars completing surveys in Waves 5–7 of the MABEL study, a longitudinal survey of Australian doctors [8]. Every practicing clinician in Australia was invited to participate in the first wave of the study in 2008, and yearly all new doctors in the workforce are invited to participate. The study includes a cohort of specialists in training, and the response rate from the initial cohort was 20.6% with further waves of the study showing similar response rates.

The proportion of registrars with postgraduate qualifications in each specialty, as well as the type of qualification (PhD, Master’s degree, postgraduate diploma/ certificate, research degree) was compared to overall rates and assessed using one sample z-test for proportions with continuity correction.

MABEL was approved by the University of Melbourne’s Faculty of Business and Economics Human Ethics Advisory Group and the Monash University Standing Committee on Ethics in Research Involving Humans.

## Results

Overall, 995 registrars representing 13 specialties (excluding general practice) were included. A total of 46.7% (95% CI 43.4–49.6) of surveyed trainees held at least one qualification in addition to their basic medical degree. There was no statistically significant difference between the proportion of male or female trainees with extra qualifications (44 and 49% of the sample respectively). Table 1 presents the proportion of specialist trainees having completed extra training in addition to their medical degree. This ranged from up to three quarters (78.6%) for ophthalmology and 67.5% of paediatric trainees to one third (34.8%) of psychiatric trainees.

**Table 1:** Postgraduate qualifications of Australian specialist trainees 2010, 2014 MABEL waves 5–7

		Any Postgraduate or Research Qualifications		PhD		Master’s Degree		Postgraduate Diploma/Certificate	Medical School Research Degree		
Speciality	n	n	(%)	n	(%)	n	(%)	n	(%)	n	(%)
Anaesthesia	127	56	(44.1%)	3	(2.4%)	5	(3.9%)	22	(17.3%)	41	(32.3%)
Dermatology	17	7	(41.2%)	0	(0%)	3	(17.6%)	0	(0%)	6	(35.3%)
Emergency medicine	128	47	(36.7%)	0	(0%)	12	(9.4%)	24	(18.8%)	23	(18%)
Intensive care medicine	41	16	(39%)	0	(0%)	3	(7.3%)	10	(24.4%)	7	(17.1%)
Obstetrics and Gynaecology	90	49	(54.4%)	3	(3.3%)	13	(14.4%)	23	(25.6%)	31	(34.4%)
Ophthalmology	14	11	(78.6%) (95%CI 49.2–95.3)	3	(21.4%) (95%CI 4.6–50.7)	6	(42.9%) (95%CI 17.6–71.1)	1	(7.1%)	7	(50%) (95%CI 23–77)
Paediatrics and child health	114	77	(67.5%)	5	(4.4%)	13	(11.4%)	47	(41.2%) (95%CI 32.1–50.8)	38	(33.3%)
Pathology	41	15	(36.6%)	0	(0%)	1	(2.4%)	5	(12.2%)	13	(31.7%)
Physician	241	103	(42.7%)	4	(1.7%)	15	(6.2%)	23	(9.5%)	84	(34.9%)
Psychiatry	69	24	(34.8%)	2	(2.9%)	7	(10.1%)	4	(5.8%)	16	(23.2%)
Radiation oncology	13	5	(38.5%)	0	(0%)	1	(7.7%)	1	(7.7%)	4	(30.8%)
Radiology	28	14	(50%)	0	(0%)	3	(10.7%)	3	(10.7%)	9	(32.1%)
Surgery	72	41	(56.9%)	4	(5.6%)	18	(25%)	14	(19.4%)	23	(31.9%)
Total	995	465	(46.7%) (95%CI 43.6–49.8)	24	(2.4%) (95%CI 1.4–3.3)	100	(10.1%) (95% CI 8.2–11.9)	177	(17.8%) (95%CI 15.4–20.1)	302	(30.4%) (95%CI 27.5–33.2)

On average, 2.4% of all trainees held a PhD (95% CI 1.4–3.3%), the highest proportion among ophthalmology trainees (21.4%). One in 10 trainees completed a Master’s degree with ophthalmology (42.9%) and surgical (25.1%) trainees most likely to hold this degree.

Postgraduate diplomas/ certificates were most common for paediatric trainees (41.2%) (95% CI 32.1–50.8).

Of surgical trainees, 57% (95% CI 44.7–68.6) held additional postgraduate or research qualifications. Master’s degrees and medical school research degrees were the most common postgraduate qualifications attained by surgeons.

## Discussion

This study sought to investigate the additional qualifications obtained by specialist registrars in Australia and reveals that almost half of trainees (46.7%) surveyed have completed at least one post graduate qualification in addition to their basic medical degree, the highest being among ophthalmology, paediatric and surgical trainees. Anecdotally there has been evidence that medical graduates were completing additional qualifications to better their chances of speciality selection, however there was no evidence to suggest the magnitude of proportion of speciality registrars undertaking additional credentialing [9]. It is now clear that Australian specialists in training are highly qualified, with degrees that are not strictly required for clinical practice. Candidates are incentivised to complete these degrees however, as they are scored when assessing CVs for selection.

The differing rates of additional qualifications and type of qualifications may reflect availability of courses, competition for training program entry and apparent specialist college preference for candidates with certain qualifications. For example, the Diploma of Obstetrics and Gynaecology offered by the Royal Australian and New Zealand College of Obstetricians and Gynaecologists (RANZCOG), is commonly undertaken by prospective trainees to demonstrate their interest in the specialty. Similarly, Diplomas of Child Health are frequently undertaken by potential paediatric trainees. This type of diploma qualification does not exist for most other specialities.

The Master of Medicine (Ophthalmic Science) is a long-established degree designed to prepare potential candidates for Royal Australian and New Zealand College Ophthalmology primary examinations and to demonstrate a candidate’s interest in the specialty. Indeed, the course outcomes include ‘to assist primarily medical graduates applying for a position in a Royal Australian and New Zealand College of Ophthalmologists (RANZCO) recognised training program’ [10]. Again, while not unique, few other specialties have University coursework tailored to selection for specialty training and primary examinations.

Our study highlights the number of additional qualifications held by medical specialists has markedly increased. In 2003 2.8% of fully qualified specialists surveyed by McGrath et al. held a PhD or Master’s degree [11] compared to the 12.5% of specialist registrars in training in 2010 to 2014. Whilst not directly comparable, as the 2003 data focused on fully qualified rather than training specialists, this data shows that the number of additional qualifications held by new trainees has increased when compared to their fully qualified peers. This may be explained by factors such as the development of specific pathways to training positions, changes to the selection criteria of candidates and the scoring of postgraduate degrees in CV assessment and increased competition for training positions.

This study shows 30% of specialist trainees have medical school research degrees which are designed to give students the opportunity to gain research experience in preparation for academic or clinical careers. These degrees, such as the BMedSci (Hons) are widely available at many Australian medical schools [12]. The move away from Bachelor of Medicine degrees to the MD in Australia, is likely to reduce the number of these degrees in the future, as they are not offered in MD programs which include a research project.

Obtaining postgraduate qualifications prior to selection for specialist training increases the costs of, and frequently the time required to complete, specialist training and may come at the cost of gaining general medical or surgical experience. Although, proponents argue that additional degrees improve the standard of trainees and accelerate knowledge acquisition, critical thinking skills and provide opportunities to improve non-technical skills such as research, communication and clinical teaching, these degrees are not pre-requisites for clinical practice, and indeed there is no research to suggest that they improve the clinical skills of candidates. The costs of obtaining these degrees can be significant. The Master of Surgical Science course at Melbourne University requires 3 years of part time study and costs $46,556 [13]. Courses such as the Diploma in Child Health/International Postgraduate Certificate (now the Sydney Child Health Program) can be completed in 1-year part time, and costs $3550 [14]. While many of these programs are offered online, most require some attendance, invariably in major cities which may disadvantage rural candidate’s ability to undertake such study.

There are some limitations of our study, due to the small sample size of some specialist subgroups and the fact that data is based on survey respondents, who may not be representative of the profession. The initial cohort of specialist registrars had a 20.6% response rate, and although this varied between waves, the total number of assessed trainees in this study (995) represents approximately 20% of the population of specialists in training [8]. A further limitation is a lack of data pertaining to when additional qualifications were obtained. Medical school research degrees must be completed prior to specialist training program commencement, and vocational Master programs such as those in Ophthalmic Science and Paediatrics require a medical degree for entry. These courses are often designed to assist applicants to be selected for the program as discussed above and are likely completed pre-selection but it still unclear as to exactly when PhDs and some Master’s degrees were completed. This information is not available in the dataset. On balance, it is likely that most of the sample surveyed completed their additional qualifications prior to or during specialty training. The PhD data also reflects local trends. Traditionally, physicians complete their PhD (in their area of interest), either after finishing, or towards completion of, physician training. In contrast, ophthalmology applicants benefitted from presenting with a completed PhD.

Entry into specialty training is gained by a combination of interview, referees reports and qualifications. Anecdotally medical graduates are undertaking qualifications to increase chances of selection to speciality training, and this study has revealed the highly qualified nature of Australian speciality graduates. This study only addresses the additional tertiary qualifications obtained by specialists in training and provides useful information for aspiring specialists. Further research would benefit from the inclusion of specific questions in future MABEL waves concerning the clinical experience of specialists in training, including non-accredited training time and overseas training prior to the commencement of specialist training, and specific data as to the nature and timing of tertiary degrees obtained. Similar data is also required for general practice trainees.

## Conclusion

This study provides information about the types of qualifications held by current specialists in training and shows the highly qualified nature of Australian specialist trainees. Australian specialist trainees are more qualified than specialists who trained in the past. It is likely that this is in part driven by increasing competition for specialty training positions where these qualifications may improve the chances of gaining training program entry. Additional qualifications are both time consuming and costly, are not mandatory for clinical practice and it is unclear whether they confer any benefit to the qualified specialist in comparison to further surgical or medical experience.

## Data Availability

The data that support the findings of this study are available from Medicine in Australia: Balancing Employment and Life (MABEL) but restrictions apply to the availability of this data, which was used under license for the current study, and so are not publicly available. Data is available from the authors upon reasonable request and with permission of MABEL.

## Abbreviations

MABEL: Medicine in Australia: Balancing Employment and Life
RANZCO: Royal Australian and New Zealand College of Ophthalmologists
RANZCOG: Royal Australian and New Zealand College of Obstetricians and Gynaecologists
